# The Metabolic Features of Osteoblasts: Implications for Multiple Myeloma (MM) Bone Disease

**DOI:** 10.3390/ijms24054893

**Published:** 2023-03-03

**Authors:** Oxana Lungu, Denise Toscani, Jessica Burroughs-Garcia, Nicola Giuliani

**Affiliations:** 1 Department of Medicine and Surgery, University of Parma, 43126 Parma, Italy; 2 Hematology, Azienda Ospedaliero-Universitaria di Parma, 43126 Parma, Italy

**Keywords:** osteoblasts, metabolism, glutamine, multiple myeloma, bone disease

## Abstract

The study of osteoblast (OB) metabolism has recently received increased attention due to the considerable amount of energy used during the bone remodeling process. In addition to glucose, the main nutrient for the osteoblast lineages, recent data highlight the importance of amino acid and fatty acid metabolism in providing the fuel necessary for the proper functioning of OBs. Among the amino acids, it has been reported that OBs are largely dependent on glutamine (Gln) for their differentiation and activity. In this review, we describe the main metabolic pathways governing OBs’ fate and functions, both in physiological and pathological malignant conditions. In particular, we focus on multiple myeloma (MM) bone disease, which is characterized by a severe imbalance in OB differentiation due to the presence of malignant plasma cells into the bone microenvironment. Here, we describe the most important metabolic alterations involved in the inhibition of OB formation and activity in MM patients.

## 1. Introduction

Osteoblasts (OBs) are bone-forming cells that originate from multipotent mesenchymal stem cells (MSCs) located in the bone marrow (BM). OBs are essential for skeletal development and fracture repair as they are the only cells able to form new bone in vertebrates [1]. Their differentiation from MSCs is triggered by the expression of specific genes, which are subsequentially regulated by pro-osteogenic pathways, including the wingless/int1 (WNT)/β-catenin signaling pathway and runt-related transcription factor 2 (RUNX2) [2]. Once differentiated, mature OBs can become osteocytes, which are incorporated into the bone matrix [3]. During bone remodeling, OBs cooperate with osteoclasts (OCs) to ensure that bone formation is coupled with bone resorption [4]. Given their primary role in maintaining bone integrity and health, OBs need a substantial amount of energy. Glucose represents one of the main fuels that sustain energy production, mainly through glycolysis, although metabolic plasticity features characterize OB differentiation [5]. In addition to glucose, fatty acids and amino acids are additional sources to sustain energy metabolism in OBs. 

Studies into amino acid metabolism have received high attention, in particular those regarding glutamine (Gln). This non-essential amino acid represents one of the main fuels for OBs since its uptake and metabolism increase during OB differentiation [1,6,7]. Several Gln transporters and enzymes have been implicated in the regulation of bone formation, opening a new potential therapeutic scenario for malignant bone-related diseases. Among these, multiple myeloma (MM) represents the main hematological malignancy condition characterized by severe uncoupled and unbalanced bone remodeling [8]. 

In this context, malignant plasma cells (PCs) accumulate and proliferate in the bone marrow, disrupting the physiological bone remodeling process, leading to impaired OB differentiation and osteolytic lesions. Recent studies highlight the importance of the alterations of MM metabolism in controlling OB functions within the microenvironment. In particular, the Gln-deprived microenvironment characteristic of MM inhibits the OB differentiation of MSC by altering the expression of OB markers, pointing to a clear involvement of amino acid metabolism in MM bone disease [7]. 

Here, we discuss the main pathways involved in OB differentiation in physiological conditions and the involvement of glucose, fatty acids, and amino acids in sustaining their bioenergetic demand and differentiation. Additionally, we focus on the involvement of OBs in bone disease, with a special emphasis on MM, and the main metabolic alterations responsible for their dysregulation in MM bone disease. 

## 2. From Bone Marrow Mesenchymal Stromal Cells to Osteoblasts

The BM niche is a synergetic network of several cellular populations that supports the hematopoietic stem cells (HSCs). In 1968, Friedenstein et al. demonstrated that not only HSCs but also MSCs reside in the BM [9]. MSCs are multipotent cells characterized by the ability to give rise to OBs, adipocytes, or chondrocytes [10]. The OBs play a crucial role in the formation and preservation of the bone architecture; these cells are responsible for the deposition of bone matrix and the regulation of OCs. During the course of development, OBs can have three possible fates: they can become a bone-lining cell or an osteocyte, or undergo apoptosis [3]. 

The osteoblastic differentiation process is deeply controlled by numerous molecular factors present in the BM microenvironment, and it occurs in a specific chronological sequence. The pathways and factors principally involved in osteoblastic differentiation will be outlined below.

RUNX2 is the main transcription factor crucial for OB differentiation. It belongs to the RUNX family, which consists of RUNX1, RUNX2, and RUNX3 [11]. In OB precursors, RUNX2 interacts with CBFβ, a co-transcription factor, and regulates the expression of osterix (OSX), as well as that of bone matrix genes including type I collagen (*COL1A1*), *COL1A2*, osteopontin (*OPN*), bone sialoprotein (*BSP*), and osteocalcin (*BGLAP2*), inducing OB maturation [12]. Furthermore, RUNX2 enhances osteoblastogenesis by modulating the expression of the hedgehog (*HH*) signaling pathway, fibroblast growth factor (*FGF*), *WNT* pathway, parathyroid hormone (*PTH*), and distal-less homeobox 5 (*DLX5*) genes [13]. Therefore, RUNX2 is a key cross-functional transcription factor, and CBFβ regulates bone structure by modulating the stability and activity of RUNX family proteins. It has also been reported that the interaction of RUNX2 and transforming growth factor-beta (TGF-β)/bone morphogenic protein (BMP) signaling induces the expression of OB-specific genes [14]. Signal transduction by TGF-β/BMPs occurs through canonical SMAD-dependent pathways (TGF-β/BMP ligands, receptors, and SMAD) and non-canonical SMAD-independent signaling pathways (the p38 mitogen-activated protein kinase, MAPK pathway) [15]. The binding of BMPs to their receptors determines the phosphorylation of SMAD1, SMAD5, or SMAD8, which, forming a complex with SMAD4, enter the nucleus and regulate gene expression, improving the function of mature OBs. BMP-2 significantly increases osteocalcin levels, while BMP-7 induces the expression of osteoblastic markers, such as alkaline phosphatase (ALP) activity, and increases calcium mineralization [16].

WNT-family proteins secreted by cells can induce cellular mechanisms through the activation of a trans-membrane complex composed of FZD (frizzled) receptors and LRP5/6 co-receptors. Once the pathway is activated, intracellular proteins and transcription factors regulate proliferation, migration, and gene expression [17]. WNTs stimulate at least three distinct signaling cascades: the canonical WNT/β-catenin pathway and two β-catenin independent non-canonical pathways (WNT/Ca^2+^ and WNT/planar polarization) [18]. Among these, the canonical pathway is of high relevance in osteoblastic differentiation. β-Catenin inactivation results from ubiquitination and proteosomal degradation due to a lack of binding between WNT proteins and the FZD receptor. The activation of the pathway, on the other hand, promotes β-catenin accumulation in the cytoplasm by inhibiting the formation of the degradation complex. β-catenin next translocates into the nucleus where it regulates and induces the expression of several genes involved in OB differentiation [19]. Furthermore, WNT/β-catenin signaling promotes the expression of osteoprotegerin (OPG) in mature OBs, which in turn suppresses osteoclastogenesis [20]. Sclerostin, encoded by the *SOST* gene, inhibits canonical WNT signaling by binding to LRP5/6 and preventing it from binding to the FZD receptor [21]. It is widely expressed by osteocytes and negatively regulates the differentiation and function of OBs. Indeed, mutations in the *SOST* gene generate sclerosing bone disorders such as sclerosteosis and Van Buchem disease [22]. Dickkopf-1 (DKK-1) is another WNT signaling antagonist that is highly expressed in bone tissue and, by binding to the LRP5/6 receptor, leads to the internalization of the complex and inhibition of cell signaling [23]. The intricate interaction between these components leads to the regulation of RUNX2 and OSX expression while inhibiting the expression of adipogenic transcription factors and blocking preadipocyte differentiation [24].

In addition to the other factors described above, PTH, produced by parathyroid gland cells, also influences OB differentiation through the stimulation of proteins involved in bone formation, such as insulin-like growth factor (IGF-1), FGF-2, and WNT/β-catenin. Besides increasing the number of OBs, PTH also promotes higher matrix deposition and suppresses apoptosis.

The main PTH-stimulated physiological pathway involves the activation of the PTH1 receptor (PTH1R) and further stimulation of cAMP, which brings about the phosphorylation and activation of protein kinase A (PKA) [25].

NOTCH activation in the early stages of OB differentiation reduces the maturation of cells able to synthesize a mineralized matrix, while its induction in mature cells prevents further differentiation and results in an accumulation of abnormal OBs [26]. These effects are most likely mediated by the downregulation of RUNX2 transcription and decreased WNT signaling. Indeed, it has been shown that in cells overexpressing NOTCH, cytosolic β-catenin levels and the stimulation of ALP activity by WNT3 are suppressed by NOTCH [27]. While some studies report that NOTCH signaling inhibits OB formation [27,28], others suggest opposite phenotype [29].

FGF signaling has different roles in OB lineage cells. FGFs have both autocrine and paracrine functions on tumor and stromal cells, and by binding tyrosine kinase receptors (FGFRs), they activate multiple signaling pathways, including RAS-MAPK, PI3K-AKT, and canonical WNT signaling [30]. These pathways regulate pre-osteoblastic proliferation and osteoblastic differentiation, including the function of mature OBs [31]. 

**Figure 1:**
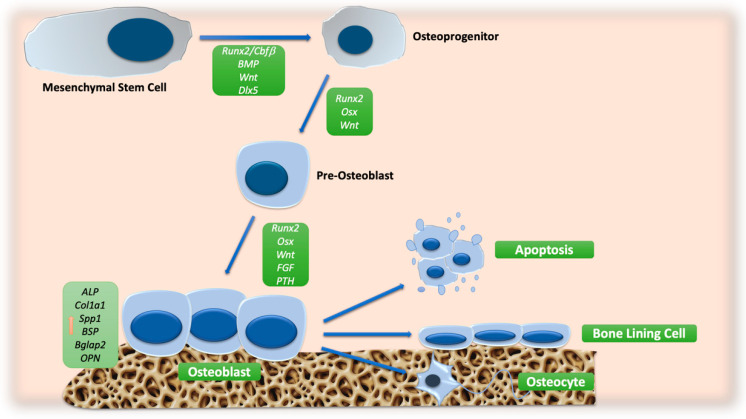
Schematic representation of molecular signals involved in OB differentiation. The orange arrow indicates the increase in the expression of osteoblast markers. Abbreviations: ALP: alkaline phosphatase; BGLAP2: osteocalcin; BMP: bone morphogenic protein; BSP: bone sialoprotein; CBFβ: core-binding factor β; COL1A1: collagen type 1; DLX5: distal-less homeobox 5; FGF: fibroblast growth factor; OPN: osteopontin; OSX: osterix; PTH: parathyroid hormone; RUNX2: runt-related transcription factor 2; SPP1: secreted phosphoprotein 1; WNT: wingless/int.

An overview of the main signaling pathway involved in OB differentiation is reported in Figure 1. 

## 3. Bone Remodeling Cycle

**Figure 2:**
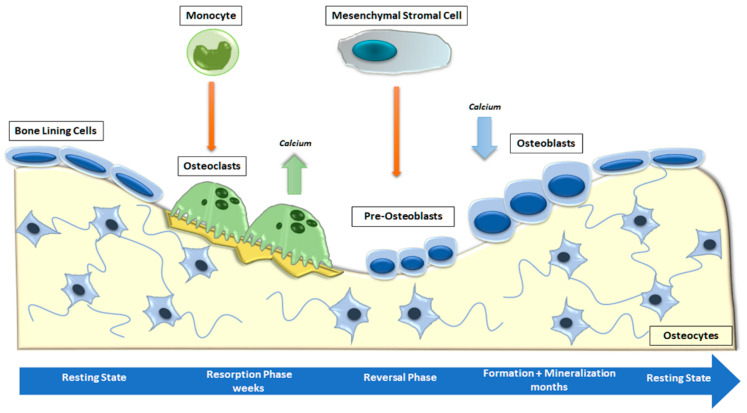
Phases of physiological bone remodeling. In a well-balanced and coupled system, bone remodeling starts with OC activation and bone resorption and ends with OB formation. The complete cycle consists of the activation, resorption, reversion, formation, and mineralization stages and is interspersed with the resting phase.

Bone is a metabolically active connective tissue presenting four types of cells: OBs (cells that form new bone), bone-lining cells that cover the surface of bone, osteocytes, and OCs (cells that destroy bone). Its function is to ensure the shape, protection, and sustenance of body structures and to facilitate locomotion [4]. Bone is mostly composed of hydroxyapatite crystals and several types of extracellular matrix proteins, including COL1A1, osteocalcin, osteonectin, secreted phosphoprotein 1 (SPP1), Integrin-Binding Sialoprotein (IBSP) and proteoglycans. Most of these bone matrix proteins are secreted and deposited by polarized mature OBs [32]. The combined activity of the cells listed above forms the temporary anatomical structure called the Basic Multicellular Unit (BMU). Within the BMU, cellular activity is coupled, which means that the amount of bone destroyed by OCs is equal to the amount of bone formed by OBs. Osteocytes, which are former OBs distributed throughout the mineralized bone matrix, perceive and react to mechanical and hormonal stimuli and coordinate the function of OBs and OCs [33]. Bone remodeling is a continuous cycle that occurs at targeted sites in the skeleton due to mechanical and metabolic influences. The cycle begins with the formation and activation of OCs that mediate bone resorption; this process is followed by a long period of OB-mediated bone matrix formation, culminating in matrix mineralization [34] (Figure 2). 

### 3.1. Initiation Phase

The process starts with the recruitment of hemopoietic myelomonocytic precursors by chemotactic cytokines released from nearby cells. Monocyte chemoattractant protein-1 (MCP-1, also known as CCL2) is secreted by OBs and is one of the most important cytokines in the recruitment of OC precursors [35]. Another chemokine produced by bone vascular endothelial cells and BM stromal cells, stromal cell-derived factor 1 (SDF-1), binds to OC precursors expressing the chemokine receptor CXCR4 and induces the expression of matrix metallopeptidase 9 (MMP-9) since the collagen-rich bone matrix is degraded by proteases such as cathepsin K and matrix metalloproteinases [36]. The osteoclastogenic factors expressed by OB lineage cells include receptor activator of nuclear factor-B ligand (RANKL) and macrophage colony-stimulating factor (M-CSF). RANKL interacts with its monocyte-expressed RANK receptor, inducing the activation of tumor necrosis factor (TNF) receptor-associated factor 6 (TRAF6). In turn, TRAF6 stimulates NF-ĸB and MAPKs, such as p38, which are responsible for the activation of transcription factors such as AP-1 (c-Fos) and NFATc1 [37]. Furthermore, OBs are involved in the regulation of osteoclastogenesis through modulation of the RANKL/OPG ratio. Indeed, OBs synthesize OPG, a soluble decoy receptor for RANKL, which is involved in the competitive inhibition of RANK/RANKL signaling, thereby preventing RANK and OC activation in several bone remodeling diseases [38]. Osteocytes are the cells that probably determine which bone surface will be resorbed by OCs. They are interconnected in the bone matrix through a network of osteocyte canaliculi-containing osteocyte dendritic processes [39]. It has been proposed that microfractures and loss of mechanical loading in bone are first detected by osteocytes, which then trigger OC differentiation [40].

### 3.2. Transition Phase 

During the transition phase, also known as the reversal phase, bone resorption is coupled with bone formation. OCs stimulate the differentiation of OBs, thus enabling bone growth in the gaps of bone resorption. While bone formation is activated, the high amount of extracellular calcium released during resorption induces the apoptosis of OCs via Bim/caspase-3 or through the Fas/Fas ligand pathway [41]. In addition, connexin-mediated communication between OBs stimulates the differentiation and activation of OBs themselves. The presence of connexin was also observed in OCs. These data suggest that due to the presence of gap junctions between OCs and OBs, intercellular communication can happen while the cells are next to each other [42]. OC–OB communication can also occur without cell–to-cell contact. Indeed, following resorption, growth factors such as TGF-β, BMPs and IGF-II are released from the bone matrix, activating osteoblastic bone formation [43]. New bone formation can be described in two stages: first, OBs form and secrete an osteoid matrix rich in COL1; second, OBs regulate osteoid mineralization [44]. During the mineralization, hydroxyapatite crystals are settled between collagen fibrils. This process is complicated, and how it is controlled is not completely comprehended [44]. New bone formation is regulated by the local and systemic phosphate/calcium levels and by inhibitors of mineralization, including pyrophosphate and non-collagenous proteins such as SPP1 [45]. Tissue non-specific ALP and ectonucleotide pyrophosphatase activities are the main factors that determine the inorganic pyrophosphate to phosphate ratio, which represents a key regulator of mineralization [45].

### 3.3. Termination Phase 

Once mineralization is over, OBs undergo apoptosis, become bone-lining cells, or remain trapped in the bone matrix and terminally differentiate into osteocytes. Osteocytes play a key role in signaling the end of remodeling through the secretion of osteogenesis antagonists, particularly WNT signaling pathway antagonists, such as sclerostin [46]. The most important pathways that determine the balance between resorption and bone formation are RANKL/RANK/OPG [47] and WNT signaling [48]. An altered expression of RANKL and OPG is a driving mechanism behind bone metastasis [49], cancer treatment-induced bone loss, and osteolysis in patients with MM [50]. At this terminal stage, OC differentiation is suppressed, probably through OPG produced by OBs. OC–OB interaction may induce NOTCH signaling in OBs, resulting in increased OPG production that inhibits RANK signaling and, thus, osteoclastogenesis [51]. In conclusion, communication between OBs and OCs at various stages of their differentiation is crucial for bone remodeling cycles. The beginning of fusion, attachment, activity, and apoptosis in OCs is controlled by cells of the OB lineage, including lining cells, pre-OB, and osteocytes. On the other hand, OCs control the level of OB activity through the release of factors within the matrix. Thus, it is through these complex intercellular communication pathways that bone can respond effectively to hormonal, mechanical, and inflammatory stimuli, providing a strong and versatile structure for the human body.

## 4. Osteoblast Metabolism

The efficient functioning of OBs needs considerable energy production, particularly during stages of new bone formation and remodeling. In humans, the remodeling process requires about 120 days, and the bones of the skeleton are completely remodeled every 10 years [52]. Bone mass preservation during the remodeling process is crucial for skeletal strength and calcium homeostasis. Since both modeling and remodeling processes necessitate the synthesis of collagen and various matrix proteins by OBs, they use a considerable amount of energy in the form of adenosine triphosphate (ATP) [52]. Very early studies showed that an increased number of mitochondria characterized mature OBs [53,54]. Later, several papers demonstrated OBs’ metabolic plasticity with increased ATP production during differentiation [5].

**Figure 3:**
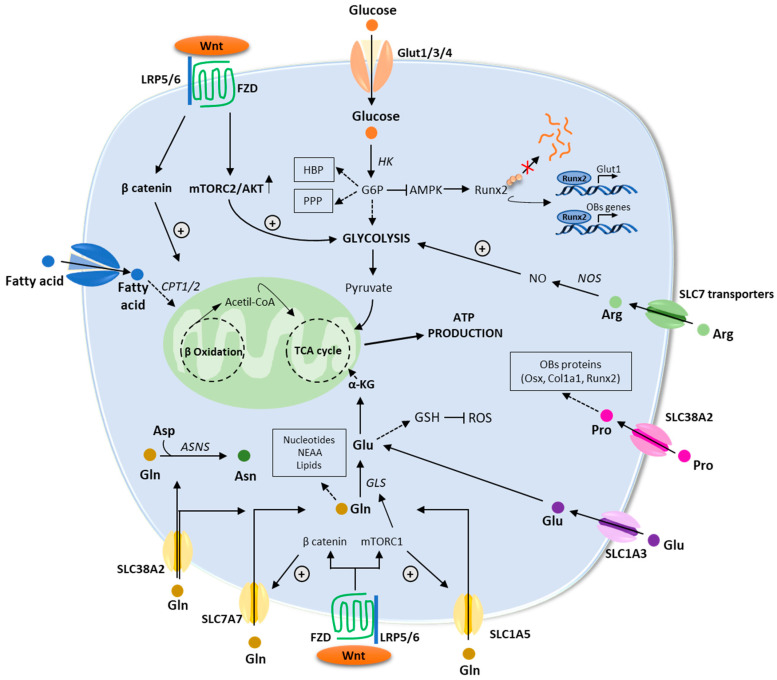
An overview of the energetic metabolism of osteoblasts. Schematic description of the main metabolic pathways including glycolysis, fatty acid, and amino acids metabolism. Enzymes are in italic. Abbreviations: αKG: α-ketoglutarate; AMPK: AMP-activated protein kinase; Arg: arginine; Asn: asparagine; ASNS: asparagine synthetase; Asp: aspartate; ATP: adenosine triphosphate; CPT1/2: carnitine palmitoyltransferase 1/2; FZD: frizzled; GLS: glutaminase; GSH: glutathione; GLUT1/3/4: glucose transporter 1/3/4; G6P: glucose-6-phosphate; HBP: hexosamine biosynthetic pathway; HK: hexokinase; LRP5/6: low-density lipoprotein receptor–related protein 5 or 6; mTORC1/2: mammalian target of rapamycin complex 1/2; PPP: pentose phosphate pathway; ROS: reactive oxygen species; RUNX2: runt-related transcription factor; TCA: tricarboxylic acid; WNT: wingless/int1.

Recent studies have revealed that the WNT pathway directly reprograms cellular metabolism by stimulating aerobic glycolysis, fatty acid oxidation, and Gln catabolism in OB lineage cells [55]. WNT-mammalian target of rapamycin complex-1 (mTOR) signaling increases protein levels of key enzymes implicated in glucose and Gln metabolism [55]. Furthermore, mRNA levels for genes implicated in fatty acid oxidation increased in response to β-catenin activation [52]. In the next paragraphs, we will provide an overview of the main metabolic pathways regulating OB functions (Figure 3). 

### 4.1. Glucose Metabolism

One of the most significant fuel substrates for OBs is glucose. It is carried into cells via glucose transporters (GLUTs) in a process known as facilitated diffusion that does not consume energy. [56]. In OBs, glucose transporter 1 (GLUT1), encoded by SLC2A1, appears to be the major glucose transporter, although GLUT3 and GLUT4 are also expressed [57]. Glucose transport through GLUT1 has been seen to stimulate the differentiation of OBs and, consequently, bone formation by blocking the proteasomal degradation of RUNX2 [58] and by stimulating mTORC1-mediated protein synthesis to enhance collagen matrix production [59]. In fact, a GLUT1-deficient mouse model in osteolineage cells showed altered OB differentiation and formation compared to wild-type animals [58]. Once internalized by the cell, the enzyme hexokinase (HK) phosphorylates glucose to form glucose-6-phosphate (G6P). Next, G6P can be catabolized through multiple pathways, such as glycolysis, the hexosamine biosynthetic pathway (HBP), and the pentose phosphate pathway (PPP) [60]. Via glycolysis, one molecule of glucose is converted into two molecules of pyruvate and two of adenosine triphosphate (ATP). Pyruvate is transported to the mitochondrion, where it is decarboxylated and oxidized to acetyl-CoA. Acetyl-CoA subsequently enters the tricarboxylic acid cycle (TCA) for mitochondrial respiration. Throughout this multistep process, three moles of NADH and one mole of FADH2 are generated. Such molecules are required to supplement oxidative phosphorylation (OXPHOS) and guide the electron transport chain, leading to ATP formation [61]. As stated before, during differentiation, OBs and their progenitors undergo profound energetic reprogramming. Guntur et al. showed that mouse OB progenitors mainly rely on glycolysis to generate ATP, while oxidative phosphorylation is preferred after the onset of differentiation and matrix production. After mineralization, mature OBs prefer glycolysis [5]. Supporting these observations, metabolic tracing studies revealed that in mature OBs, most of the glucose is converted to lactate and ATP is generated mostly via glycolysis [62,63]. 

The reason why OBs choose aerobic glycolysis is currently not fully comprehended. From a bioenergetic point of view, aerobic glycolysis has a lower efficiency in terms of ATP production than metabolism via TCA and OXPHOS [64]. Tumor cells show related metabolic reprogramming, known as the Warburg Effect, which is supposed to provide amino acids, nucleotides, and lipids necessary to sustain cell division. Furthermore, enhanced aerobic glycolysis could help reduce reactive oxygen species and also contribute to the generation of more amino acids to support protein synthesis in OBs [65]. 

WNT3A/LRP5 signaling contributes to the regulation of OB metabolism by stimulating aerobic glycolysis, a mechanism mediated by mTORC2-AKT signaling but independent from β-catenin [62]. Moreover, the metabolic rewiring seems necessary for OB differentiation since glucose shortage impairs differentiation in response to WNT3A [62]. 

PTH/PTHR1 signaling promotes bone anabolism by stimulating the expression of glycolytic enzymes and aerobic glycolysis. This mechanism is mediated by the IGF/mTOR pathway, which plays an essential role in bone metabolism [66]. Interestingly, the inhibition of glycolysis reduces the anabolic effect of PTH in mice [66], providing a possible link between bone metabolism and the anabolic effect of PTH. 

Conversely, the NOTCH pathway restricts OB differentiation by inhibiting the expression of enzymes involved in the glycolysis pathway and mitochondrial respiration in primary mesenchymal progenitors, resulting in decreased mitochondrial respiration and AMPK activity [67]. Until now, only a few signaling pathways have been proposed to regulate the energy metabolism of OBs such as HH, IGF1, and BMP. In particular, HH, by inducing IGFs, stimulates OB differentiation and mTORC/AKT signaling, thus providing a possible link between OB differentiation and energy metabolism [68]. Many BMP molecules have been implicated in the regulation of glucose metabolism by increasing glucose uptake and utilization. More importantly, BMPs indirectly control glucose metabolism by regulating the WNT, PTH, and mTOR pathways [69]. 

### 4.2. Fatty Acid Metabolism

BM fat can occupy about 70% of the available bone volume in healthy adults. This suggests that fatty acids released from triglycerides stored in the marrow may be an important source of energy to meet the energetic demands of bone formation [70]. Lipids can be acquired as free fatty acids taken up by cell surface transporters or as lipoprotein particles bound by members of the LDL receptor family. Once inside the cell, the fatty acids are transported to the mitochondria via a shuttle formed by carnitine palmitoyltransferase 1 (CPT1) situated on the outer mitochondrial membrane and CPT2 placed on the inner membrane [71]. Fatty acids are metabolized in the mitochondrial matrix via β-oxidation, which sequentially cleaves two carbons as acetyl-CoA, which then enters the TCA cycle [72]. Fatty acid oxidation provides more energy than that produced by glucose or amino acid metabolism. Compared to glycose, few studies have investigated the role of fatty acid metabolism in OBs. Several groups demonstrated that β-oxidation contributes to ATP production in bone tissue [73,74] and that the expression of CPT1 increases during OB differentiation [75]. Indeed, the inhibition of CPT1 reduces OB differentiation in vitro and bone healing in vivo. Recently, van Gastel et al. [76] showed that the scarcity of fatty acids stimulates chondrocyte differentiation rather than OB differentiation. On the contrary, when fatty acids are present, skeletal progenitors are stimulated to undergo OB differentiation, reflecting the different metabolic state of OBs compared to chondrocytes [76]. It has also been demonstrated that fatty acid utilization by OBs is under the control of WNT-LRP5 signaling. Mutant mice with *LRP5* deletion have been shown to have reduced bone mass and increased fat mass and triglyceride levels, suggesting deficient fatty acid catabolism. In fact, the expression of various enzymes of β-oxidation was reduced in primary OBs in *LRP5*-deficient mice but increased in primary OBs expressing a variant of *LRP5* (*LRP5^G171V^*) associated with increased bone mass [77]. Further studies in OB-specific β-catenin-deficient mice suggested that WNT signaling controls fatty acid catabolism through the canonical WNT-β-catenin pathway [78]. 

### 4.3. Amino Acid Metabolism

Amino acids can be used by OBs for protein synthesis, or they can be metabolized to generate energy in the form of ATP. According to their catabolic pathway, ketogenic amino acids are degraded into acetyl-CoA or acetoacetate, while glucogenic amino acids are decomposed into pyruvate or various TCA intermediates [79]. Consequently, amino acids can directly support ATP production through the TCA cycle and OXPHOS. Gln, which is a nonessential amino acid (NEAA) mostly synthesized by the enzyme Gln synthetase (GS) utilizing glutamate (Glu) and ammonia (NH3) as sources, has emerged as an important regulator of OBs as the enhanced matrix synthesis associated with bone formation raises the requirement for amino acids [80]. Gln has multiple functions in cellular metabolism, from participation in the TCA cycle to the biosynthesis of nucleotides, glutathione (GSH), and NEAA [80]. It is carried into the cells via plasma-membrane Gln transporters, including SLC1A5 (ASCT2), SLC7A7, and SLC38A2 (SNAT2) [6,7]. Under normal conditions, OBs take up Gln mainly through SLC1A5, a Na^+^-dependent transporter that can also carry asparagine, serine, threonine, and alanine [81]. Throughout the proliferation phase, OBs’ Gln uptake is controlled by the general control non-depressible 2 (GCN2) mechanism. Under conditions of high-protein synthesis such as OB proliferation and matrix production, the alpha subunit of eukaryotic translation initiation factor 2 (eIF2α) is phosphorylated by GCN2. As a result, this causes an enhanced translation of the transcription factor ATF4, which promotes the expression of amino acid transporters, including SLC1A5 [82].

Once in the cell, the enzyme glutaminase 1 (GLS1) converts Gln to Glu, releasing ammonium ions. Glu can then be used for GSH biosynthesis, an antioxidant that protects cells from oxidative damage, or can be metabolized to alpha-ketoglutarate (α-KG) by Glu dehydrogenase 1 (GLUD1 or GDH1) [83]. α-KG then enters the TCA cycle and supports the OXPHOS pathway or the reductive carboxylation pathway [84]. In addition, Glu can be recruited from the extracellular compartment through the SLC1A3 transporter [85]. Besides the SLC1A3 transporter, OBs express N-methyl-D-aspartate Glu receptor (NMDAR). In vitro studies have shown that Glu can stimulate OB differentiation via NMDA or α-amino-3-hydroxy-5-methyl-4-isoxazolepropionic acid (AMPA) receptors [86]. 

GLS inhibition using the Bis-2-(5-phenylacetamido-1,3,4-thiadiazol-2-yl)ethyl sulfide (BPTES) significantly reduced intracellular Glu and αKG, but had no impact on other products of Gln metabolism, suggesting that Gln carbon is mainly used to provide αKG, which is involved in amino acid biosynthesis in skeletal stem cells (SSCs) [1].

BM stromal cells (BMSCs) consume a large quantity of Gln when they differentiate into OBs, but not into adipocytes. It has been reported that Gln increases the activity of GLS and GDH via the mTOR/S6 and MAPK signaling pathways, thereby promoting cell proliferation [87]. Furthermore, the WNT/mTORC1 pathway promotes the expression of genes involved in protein anabolism. The mTORC1-mediated increase in protein synthesis leads to higher uptake of Gln to generate more energy through the TCA cycle in order to overcome the energy deficit that occurs during OB differentiation [88]. The activation of β-catenin by WNT stimulates Gln transport via SLC7A7, while mTORC1 controls basal Gln uptake by SLC1A5 [6]. Since the OB progenitors proliferate rapidly into mature OBs able to synthesize bone matrix, the differentiation process is characterized by the increased consumption of Gln. Indeed, it has been seen that when OBs are stimulated to mineralize, glucose is not sufficient to meet their energy requirements. Only when cells are supplemented with glucose and Gln does the degree of mineralization increase [89]. Additionally, the genetic inhibition of Gln metabolism in SSCs has been shown to lead to a reduction in bone mass due to a decrease in OBs, and GLS-deficient OBs exhibit reduced bone formation [1]. Interestingly, the administration of PTH stimulates Gln uptake by inducing SLC1A5 and GLS1-dependent Gln catabolism in mice. The genetic deletion of *GLS1* in mice inhibits the bone anabolic effect of PTH. 

Beyond Gln, some other amino acids are implicated in supporting the elevated anabolic demands of OBs. A recent study reported an important role for the NEAA proline (Pro). Most of the Pro is taken up via SLC38A2 and incorporated directly into OB-associated proteins, such as RUNX2, OSX, and COL1Al [90]. Its consumption has been demonstrated to increase during osteogenic differentiation, while the deletion of *SLC38A2* in *OSX*-expressing cells has a negative impact on OB differentiation and bone development [90].

Another amino acid implicated in protein synthesis and the regulation of gene expression is methionine. The dietary restriction of this essential amino acid (EAA) has been seen to negatively impact bone properties, particularly OB differentiation [91]. However, the mechanism of these metabolic abnormalities has yet to be elucidated.

Arginine (Arg) is a conditional EAA that can be taken up mainly through the SLC7 transporters. Arg is implicated in the synthesis of nitric oxide (NO), which consequently increases glycolysis and OB differentiation. In addition, the loss of arginosuccinate lyase (ASL) in OBs, an enzyme that generates Arg and fumarate, causes a reduction in bone mass [92]. 

The tryptophan degradation pathway produces kynurenine metabolites that are related to bone mass loss in patients with osteoporosis. Indeed, kynurenine administration in adult mice has been seen to reduce bone mass [93,94].

All these studies indicate that OBs and progenitors use different amino acids in their metabolism to support anabolic functions during bone formation and mineralization.

## 5. Bone Remodeling in Pathological Conditions: The Multiple Myeloma Model 

To ensure that there is no alteration in bone mass or quality after each remodeling cycle, healthy bone remodeling needs close coupling between resorption and bone formation. Still, this essential physiological process can be hindered by multiple events, such as hormonal fluctuations associated with menopause, alterations in physical activity, drugs, age-related factors, and secondary diseases [95]. Osteoporosis is by far the most widespread disorder of bone remodeling. The pathogenesis of osteoporosis in women involves increased bone resorption resulting from changes in estrogen and FSH levels at menopause and decreased bone formation caused by a variety of factors associated with the aging process [96]. In addition to secondary forms of osteoporosis, hematologic malignancy as MM plays a very significant part. The effects of hematologic diseases on bone are not only due to the physical relationship between BM cells and bone, but also to a wide range of circulating factors that can affect bone turnover, increasing the activity of OCs and reducing OBs activity [8]. MM represents the most important pathological condition that exhibit a negative impact on bone remodeling process [8]. 

### 5.1. Pathogenesis of Multiple Myeloma Bone Disease: Osteoblasts in the Spotlight

Osteolytic bone disease is a main feature of MM leading to bone pain, skeletal-related events and, subsequently, decreased quality of life. The proliferation of malignant PCs into the BM alters the bone remodeling process, leading to uncoupled and unbalanced bone formation and resorption [97]. Both soluble factors and physical interactions are responsible for the altered bone remodeling in MM. Cell-to-cell contact between MM and BM microenvironment cells leads to enhanced production by BMSC of the osteoclastogenic factor RANKL and decreased releases of its decoy receptor OPG. As a result, the BM of MM patients is characterized by an increased RANKL/OPG ratio and enhanced OC activation. Other factors favoring osteoclastogenesis are increased into the BM by MM cells, such as chemokine (C-C motif) ligand (CCL)-3, interleukin (IL)-1, IL-3, IL-6, activin A, and TNFα [98]. 

Simultaneously, the alterations of bone resorption promote the growth of malignant PCs, supporting the vicious cycle within bone niche. MM–stromal cell interactions also impair OB formation by reducing the activity of RUNX2 in human OB progenitors [99]. Other molecules, such as IL-7 and hepatocyte growth factor (HGF), reduce RUNX2 activity contributing to OB impairment [100,101]. In the next paragraph, we will describe the main mechanisms responsible for OB suppression in MM and the metabolic implications for bone disease.

### 5.2. Osteoblast Suppression in Myeloma 

The impaired osteoblastic function in MM derives mainly from the inhibition of osteogenic differentiation of mesenchymal progenitors into mature OBs. Several studies have reported that bone remodeling in MM is uncoupled and unbalanced, with an increase in OC activation and, consequently, enhanced bone resorption and a decrease in bone formation caused by a reduction in the number and activity of OBs [102]. In addition, patients with MM have low levels of bone formation markers such as ALP and osteocalcin, while MM patients without bone disease exhibit balanced bone remodeling with normal OC differentiation and bone formation [98]. 

The main mechanism behind the OB suppression by myeloma cells is the inhibition of RUNX2 activity and expression. RUNX2 regulates the expression of various factors produced by OBs at different steps of maturation, including DKK1, WNT10, OPN, TGF-β1, BMP-4, RANKL, and OPG [103]. *RUNX2* inhibition is mediated in part by cell-to-cell contact between MM cells and OB progenitors and in part by soluble molecules produced by MM cells [104]. Adhesive interactions between malignant PCs and BMSCs are mediated by VLA-4 (α4β1 integrin) on MM cells and VCAM-1 present on BMSCs [105], while soluble factors include soluble crest-related protein (sFRP), DKK1, CCL-3, IL-7, activin A, and TNF-α. It has been demonstrated that OB-*RUNX2* deficiency induced by soluble factors released by MM cells can fuel the dissemination and progression of MM cells. Mechanistic studies have shown that OB-*RUNX2* deficiency generates a highly chemoattractive and immunosuppressive BM microenvironment, which is responsible for the recruitment and progression of MM cells to new bone sites [106]. Moreover, malignant PCs secrete WNT inhibitors that are involved in the development of osteolytic lesions by affecting OB differentiation [107,108]. Besides sclerostin and DKK1, other suppressors of the WNT signaling are secreted by MM cells. These include sFRP-2/3, which are produced by both MM cell lines, and most primary MM cells [109]. 

Sclerostin, by binding to the extracellular domain of LRP5/6 receptors, inhibits the canonical WNT pathway. As a result, β-catenin phosphorylated by a multiprotein destruction complex, in particular by GSK-3β, is degraded by proteasomal ubiquitination. Furthermore, it increases apoptosis in mature OBs by triggering the caspase pathway, resulting in the inhibition of bone formation and mineralization [110,111]. It was also demonstrated that the complete deletion of the *SOST* gene in immunocompromised SCID mice suppressed the evolution of MM-induced osteolytic lesions [112].

DKK1 is a protein belonging to the DKK family that plays a key modulatory role in bone disease in MM [113]. It is an antagonist of the WNT pathway and has a significant role in osteoblastogenesis and bone formation. DKK1 binds to LRP5/6 and the transmembrane protein Kremen1/2 to form a complex that results in LRP internalization, inhibiting the activation of the canonical WNT/β-catenin pathway [114]. It also impairs BMSCs’ differentiation into mature OBs by suppressing WNT autocrine signaling, which is required for BMP-2-mediated OB differentiation [115]. In turn, undifferentiated BMSCs release interleukin-6 (IL-6), which promotes PC growth in MM [116]. DKK1 operates synergistically with sclerostin; it deregulates the WNT pathway-mediated production of RANKL and OPG, leading to an increase in the RANKL/OPG ratio; consequently, osteoclastogenesis is indirectly enhanced [117].

Activin A, a protein belonging to the TGF-β family, recognizes the transmembrane receptor type II serine/threonine kinase (ActRIIA/B) and induces the activation of the SMAD signaling cascade, leading to the translocation of the SMAD2/3/4 complex into the nucleus. Activin A is a transcriptional factor that regulates cell proliferation, differentiation, apoptosis, and metabolism [118]. High-circulating levels of activin A have been linked to MM progression and a poor prognosis [119]. It has been observed that the communication between BMSCs and MM cells is responsible for the secretion of activin A, which exerts its inhibitory effects on OBs via the downregulation of the transcription factor *DLX5* in OB precursor cells [120].

Among the soluble factors, IL-7 and CCL-3 derived from MM cells are responsible for the inhibition of OB formation through the downregulation of *RUNX2* and *OSX*, respectively. Moreover, CCL-3 supports the survival and the homing process of MM cells into the BM niche [100,121].

### 5.3. Role of Metabolic Alterations in MM-Induced OB Suppression

Beyond the pathways involved in the osteoblastic inhibition described above, metabolic alterations in the BM microenvironment also play a crucial role in OB activity in MM patients [122]. Glucose and Gln are known to be the two main nutrients used by cancer cells to meet their energy needs [123]. Recent data indicate that malignant PCs are Gln-addicted since the cells exhibit high levels of GLS and a lack of GS expression, a feature that makes the cells particularly dependent on extracellular Gln. This characteristic modifies the physiological levels of Gln in the microenvironment with a significant impact on bone remodeling, especially on OB differentiation [124]. 

**Figure 4:**
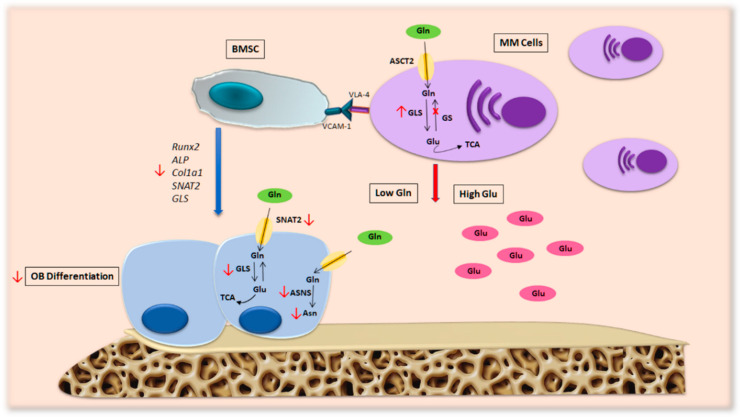
Metabolic alterations in MM patients’ microenvironment. MM cells present high levels of GLS and a lack of GS expression, a feature that makes them particularly dependent on extracellular Gln. This hallmark of malignant PCs generates a BM microenvironment characterized by low levels of Gln, which consequently alters OB formation and activity in patients with MM bone disease. Abbreviations: ALP: alkaline phosphatase; ASCT2: alanine-serine-cysteine transporter-2; ASNS: asparagine synthetase; Asn: asparagine; BMSC: bone marrow stromal cell; COL1A1: collagen type 1; Gln: glutamine; Glu: glutamate; GLS: glutaminase; GS: glutamine synthetase; MM cells: multiple myeloma cells; OB: osteoblast; RUNX2: runt-related transcription factor 2; SNAT2: sodium-dependent neutral amino acid transporter-2; TCA: tricarboxylic acid cycle; VCAM-1: vascular cell adhesion molecule-1; VLA.4: very late antigen-4.

Our group found that Gln depletion imposed by MM cells in patients’ BM compromises OB differentiation [7] (Figure 4). Analyzing the transport of Gln in MM cells and stromal cells, we saw that MM cells internalize a greater amount of Gln than stromal cells, resulting in a decrease in amino acids in the culture medium. Moreover, the differentiation potential of stromal cells decreases when they are cultured in a medium conditioned by MM and is restored by the addition of Gln. These observations suggest that MM cells generate a microenvironment characterized by low Gln levels that can affect the differentiation and activity of OBs. In fact, differentiated MSCs in the presence of a concentration of Gln mimicking the medullary plasma of patients with MM show a significant decrease in osteoblastic markers [7].

The molecular mechanism responsible for Gln’s effects is correlated with the induction of SNAT2 and GLS1 in MSCs, which are blunted by amino acid deprivation. In fact, the inhibition of GLS by CB-839 and SNAT2 using MeAIB impaired the expression of OB markers, suggesting the relevance of GLS and SLC38A2 transporter activity during osteoblastogenesis [7].

Additionally, in vitro characterization of the intracellular content of amino acids in differentiated MSC showed higher levels of the amino acid asparagine (Asn) than undifferentiated cells. Interestingly, the differentiation of OBs compromised by Gln deprivation has been reestablished by Asn integration. Analysis of the expression of asparagine synthase (ASNS), the enzyme responsible for the synthesis of Asn from Gln and aspartate (Asp), revealed an increase during differentiation, while its knockout induced a decrease in osteoblastic markers in the stromal cell line [7]. Mechanistically, Asn may be necessary to synthetize OB-specific proteins in Gln-depleted conditions. Moreover, it has been demonstrated that Asn favors the uptake of arginine and serine, thus inducing mTORC [125], which is known to stimulate OB differentiation. 

These data demonstrate that MM cells may hinder osteoblastogenesis by blocking mesenchymal Asn synthesis via Gln depletion, providing a possible metabolic mechanism behind OB inhibition in MM. Further studies are needed to elucidate the role of other amino acids in the MM bone microenvironment. 

Besides MM, the involvement of amino acid metabolism in bone homeostasis has been hypothesized in other models of bone disease. In particular, in osteoarthritis (OA), it has been demonstrated that Gln exerts positive effects on the recovery of fractured bones in standardized albino rats by achieving a positive nitrogen balance [126]. In addition, a combination of heat treatment and Gln administration has been shown to suppress OA progression in a rat model of OA [127]. Other than Gln, data indicate that L-tryptophan stimulates the proliferation of BMSCs by increasing the expression of osteocalcin and ALP. Previous studies on female rats have shown that tryptophan levels and tryptophan 2,3-dioxygenase (TDO) activity decrease in all tissues with age, correlating it with the reduction in bone mass associated with aging [128,129]. Other studies are needed to confirm the contribution of amino acid metabolism in the pathogenesis of bone-related diseases. 

## 6. Conclusions 

In the last years, different studies have investigated the role of energetic metabolism in OBs in physiological and pathological conditions. The metabolic plasticity of OBs is essential for their normal functions during bone remodeling. To date, the best-characterized source of energy for OBs is the glucose used by the cells to fulfill their high energetic demand during differentiation. Less is known about how OBs utilize fatty acids and amino acids during bone formation. 

Gln represents the main modulator of OB functions by acting at different steps of differentiation. In turn, several osteogenic molecules, such as WNT and RUNX2, regulate both glucose and Gln metabolism by increasing their uptake and catabolism. Studies also provide interesting data on the involvement of other amino acids, such as proline, arginine, and glutamate, in bone formation, although the specific mechanism remains to be investigated. Interestingly, bone-related diseases provide important evidence for the role of amino acids in regulating OB differentiation. In particular, the metabolic alterations of Gln in the MM bone niche restrict OB differentiation and function by reducing the expression of Gln transporters and enzymes, as well as the synthesis of Gln-related amino acid ASN. Lastly, from a translational perspective, targeting amino acid metabolism could represent a potential strategy to prevent bone disease. Numerous attempts have been made to target GLS and ASCT2 in MM to reduce tumor burden [130,131]. However, the results need to be finalized in vivo, and the possible effect on bone cells remains to be determined. Indeed, the supplementation of specific amino acids has been shown to improve bone health in patients with bone loss disorders [132,133]. More translational research is needed to understand the mechanism of OB metabolism regulation and, more importantly, the effectiveness of metabolic-based therapy to stimulate bone formation in MM patients. 

## Data Availability

Not applicable.
